# *In vivo *gene expression profiling of human intestinal epithelial cells: analysis by laser microdissection of formalin fixed tissues

**DOI:** 10.1186/1471-2164-9-209

**Published:** 2008-05-05

**Authors:** Michael D George, Jan Wehkamp, Robert J Kays, Christian M Leutenegger, Sadiah Sabir, Irina Grishina, Satya Dandekar, Charles L Bevins

**Affiliations:** 1 Department of Medical Microbiology and Immunology, School of Medicine, University of California, Davis CA 95616, USA; 2 Dr. Margarete Fischer-Bosch-Institute of Clinical Pharmacology, Auerbachstr. 112, 70376 Stuttgart, Germany; 3 Idexx Reference Laboratories, West Sacramento, CA 95605, USA

## Abstract

**Background:**

The small intestinal epithelium mediates vital functions of nutrient absorption and host defense. The spatial organization of the epithelial cells along the crypt-villus axis segregates them into regions of specialized function. However, the differences in transcriptional programming and the molecular machinery that governs the migration, adhesion, and differentiation of intestinal epithelial cell lineages in humans remain under-explored. To increase our understanding of these mechanisms, we have evaluated gene expression patterns of ileal epithelial cells isolated by laser capture microdissection from either the villus epithelial or crypt cell regions of healthy human small intestinal mucosa. Expression profiles in villus and crypt epithelium were determined by DNA microarray, quantitative real-time PCR, and immunohistochemistry based methods. The expression levels of selected epithelial biomarkers were also compared between gastrointestinal tissues.

**Results:**

Previously established biomarkers as well as a novel and distinct set of genes believed to be linked to epithelial cell motility, adhesion, and differentiation were found to be enriched in each of the two corresponding cell populations (GEO accession: GSE10629). Additionally, high baseline expression levels of innate antimicrobials, alpha defensin 5 (HD5) and regenerating islet-derived 3 alpha (Reg3A), were detected exclusively within the small bowel crypt, most notably in the ileum in comparison to other sites along the gastrointestinal tract.

**Conclusion:**

The elucidation of differential gene expression patterns between crypt and villus epithelial cell lineages in human ileal tissue provides novel insights into the molecular machinery that mediates their functions and spatial organization. Moreover, our findings establish an important framework of knowledge for future investigations of human gastrointestinal diseases.

## Background

Epithelial stem cell progenitors, which reside in the intestinal crypt compartment, differentiate into either secretory (goblet, enteroendocrine, Paneth) or absorptive (enterocytes) lineages, that in turn, provide a range of digestive and host defense functions within the gastrointestinal tract. Studies suggest that epithelial cell maturation, differentiation, and turn-over occur through a complex orchestration of cellular adhesion and dissociation, migration, and programmed cell death [1,2]. These events, and digestive and defense functions of the maturing cells, are likely accompanied and/or driven by wholesale alterations in gene expression. Three of the epithelial cell types migrate upward along the villus – absorptive enterocytes, goblet cells, and the far less abundant enteroendocrine cells. Absorptive enterocytes perform their nutrient absorptive and digestive functions via nutrient transport proteins and hydrolytic enzymes [3-5]. Goblet cells perform cytoprotective, anti-inflammatory, and maintenance functions through synthesis and secretion of trefoil factors and mucins [6,7]. Enteroendocrine cells are individually dispersed neuroendocrine cells that represent less than 1% of the gut epithelial cells and produce a variety of gut hormones [8]. Paneth cells, on the other hand, remain in the crypt and reside at the base, where they provide innate antimicrobial functions through production and secretion of antibiotics that combat invasion by potential pathogens and may help regulate the composition of commensal bacteria [9-12].

A range of human diseases and digestive disorders have been linked to disruptions in the proper maturation and/or terminal function of these epithelial lineages, highlighting the need to increase our knowledge of their basic physiology. Biochemical, genetic, and gene expression studies in mouse models and *in vitro *epithelial cell lines have, in recent years, provided insights towards understanding both the roles of Wnt signaling in mediating proliferation of intestinal stem cell populations [13,14], and of Wnt and Notch signaling in the differentiation of secretory populations [15-17]. Several biomarkers believed to mediate differentiation of progenitor stem cells have also been identified [18-21]. However, many details regarding the mechanisms that underlie intestinal epithelial cell differentiation and the coordination of interactions with adjacent cells and the extracellular matrix remain unknown. Moreover, similarities and differences between human intestinal epithelial cell differentiation and events observed in the mouse models and in various epithelial cell lines have not been fully established.

To address these needs, we have evaluated gene expression patterns in human ileal epithelial cells isolated by laser capture microdissection (LCMD) from either the villus or crypt epithelial cell regions of the crypt-villus axis. Genes differentially expressed in the two cellular compartments were identified through high throughput genome-scale microarray analysis and further assessed by quantitative real-time PCR and immunohistochemistry. Biomarkers previously identified *in vitro *and in animal models as either Paneth cell or villus epithelial cell specific were found to be enriched in the corresponding two cell populations in the human small intestine. Additionally, a distinct set of genes believed to be linked to epithelial cell motility, adhesion, and differentiation was found to be differentially transcribed in each population, providing novel insights into potentially important details about the molecular machinery ileal epithelial cells utilize to mediate their spatial organization and function.

## Results

### Differential expression profiling of villus and crypt epithelial cell lineages

**Figure 1:**
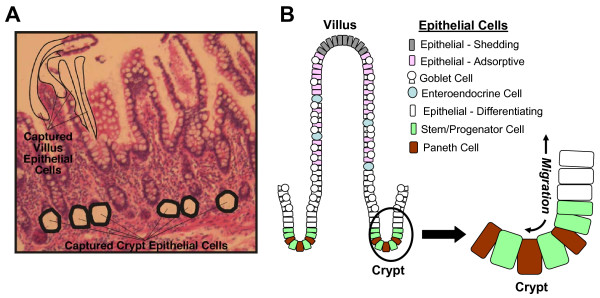
**LCMD of villus epithelial and Paneth cells**. **A. **Villus and crypt epithelial cells were excised from sections of formalin fixed human ileal tissue samples by LCMD. Areas where cells from each lineage were captured are labeled. The mRNA from the isolated cells was amplified, labeled and analyzed using both microarray and RT-PCR based methodologies. **B. **Schematic diagram of the cell types that comprise the human ileal epithelium. Epithelial cell lineages, including adsorptive, enteroendocrine, goblet, and Paneth cells are color-coded and their distribution within the epithelium is shown. The location of stem cells, shown in green, and the bidirectional cell migration is depicted in the magnified crypt region on the right.

**Figure 2:**
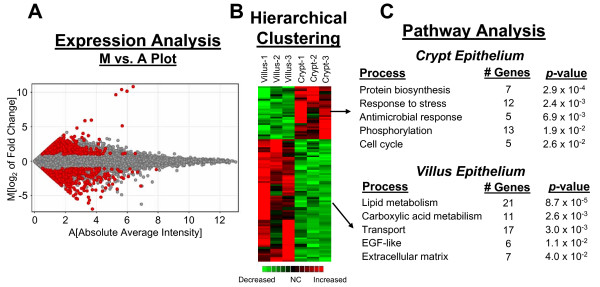
**High throughput gene expression profiling by microarray analysis**. **A. **M vs A plot showing genes identified as differentially expressed (2-fold or more, *P *value ≤ 0.05) between villus and crypt epithelial cells in red. Genes were identified through comparison of median expression levels in 3 independent paraformaldehyde-fixed ileal tissue samples from 3 different individuals. **B. **Hierarchical clustering of the genes differentially expressed (2-fold or more, *P *value ≤ 0.05). Common patterns of gene transcription were determined by non-biased clustering analysis, revealing a set of genes whose expression was enriched in each of the 2 lineages, villus and crypt epithelial cells. **C. **Pathway analysis of the differentially expressed genes reveals physiological processes that predominate the biology of villus and crypt epithelial cell lineages. Genes enriched in the 2 lineages evaluated were analyzed statistically to determine which pathways and processes were most represented each cell type. The top 5 pathways/processes implicated for each lineage are shown.

To elucidate differential gene expression profiles of small intestinal villus and crypt epithelial cells, these cell populations were excised from unstained, sectioned formalin-fixed human ileal tissue samples by LCMD (Figure 1A). The human ileal epithelial layer consists of multiple cell types (goblet, enteroendocrine, Paneth, absorptive enterocytes) that simultaneously differentiate from stem cell progenitors as they migrate toward their sites of function along the villus-crypt axis. Villus epithelial cells were identified in unstained specimens based on topographical location; crypt epithelial cells were identified based on topographical location and appearance of granular cytoplasm characteristic of Paneth cells. Following RNA extraction, linear amplification, labeling, and hybridization to DNA microarrays, the genes for which the level of detected transcripts differed by at least 2-fold (student t-test, p ≤ 0.05) between the two cell pools were identified (highlighted in red in the M vs A plot shown in Figure 2A). These genes were then hierarchically clustered to identify common patterns of differential expression (Figure 2B). Genes that were expressed predominantly in either villus or crypt epithelia were then statistically evaluated to determine which pathways and processes were most represented (Figure 2C). Genes associated with protein biosynthesis, cellular stress and antimicrobial responses, phosphorylation, and cell cycle (including Ki67) were enhanced in the crypt epithelial cells, while villus epithelial cells displayed increased expression of genes associated with lipid and carboxylic acid metabolism, transport, EGF-like activity, and extracellular matrix functions. A list of the genes differentially transcribed in these categories, their fold differences in expression between crypt and villus epithelial cells, and the statistical confidence associated with those differences, is provided in Additional file (1). The entire microarray data set is available for viewing at the Gene Expression Omnibus database (accession: GSE10629).

**Figure 3:**
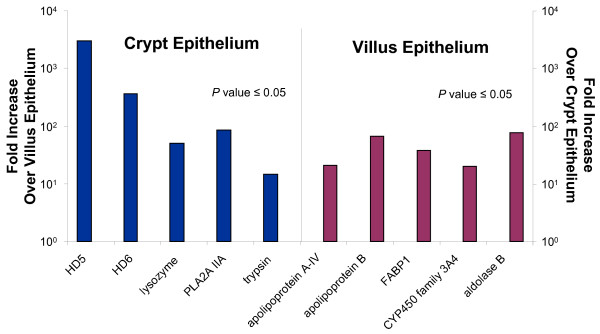
**Detection of known villus epithelial and Paneth cell biomarkers**. Relative transcriptional levels of previously characterized Paneth cell and villus epithelial biomarkers. Fold differences in expression between the crypt and villus epithelial regions are shown for HD5, HD6, trypsin, lysozyme, PLA2IIA (crypt epithelium) and carboxyesterase-2, PEP carboxykinase, aminopeptidase N, apolipoprotein A-IV, aldolase-B (villus epithelium) to demonstrate the magnitude of differential expression between the 2 cellular compartments and provide supportive evidence that the appropriate cells were captured by LCMD. Statistical validation of the differences in expression levels were determined by student t-test (*P *value ≤ 0.05).

As expected, crypt epithelial cells expressed dramatically higher levels of antimicrobial peptides characteristic of Paneth cells, including alpha-defensin-5 (HD5), HD6, lysozyme, secretory phospholipase A2, and Reg3A (Figure 3). RACE-PCR analysis confirmed that the alpha, and not the gamma, isoform of Reg3 was expressed in the human ileal tissue (data not shown), which contrasts with the nomenclature used in the mouse where Reg3γ is expressed [22-24]. Trypsin, the proteolytic enzyme associated with alpha-defensin processing [25] was also identified in the crypt. Villus epithelial cells, in contrast, displayed markedly increased mRNA levels of aldolase-B, apolipoprotein-A4, aminopeptidase-N, PEP carboxykinase, and carboxylesterase-2. The identification of these previously established intestinal epithelial biomarkers provided evidence that our isolation of the desired epithelial lineages was successful, and validated the reproducibility of the methodology using formalin-fixed paraffin-embedded samples across multiple biological replicates.

### Supportive evidence for microarray findings

**Figure 4:**
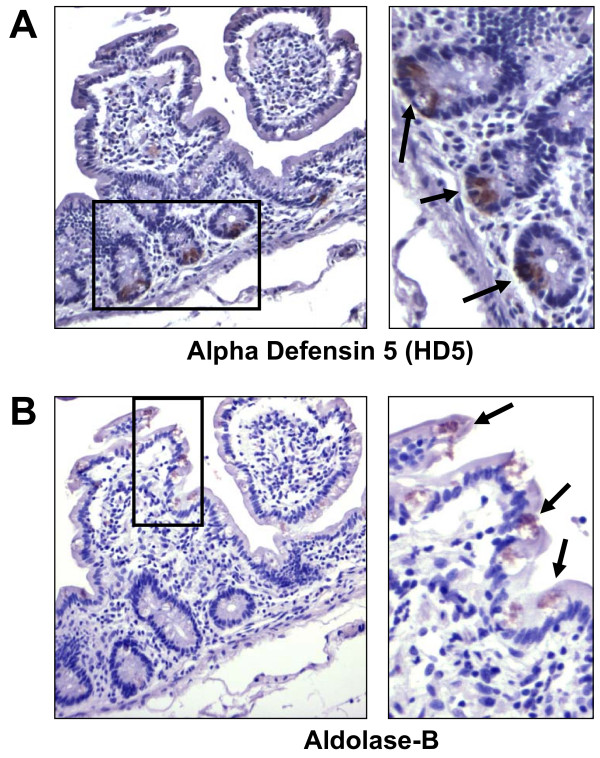
**Localization of epithelial biomarkers by immunohistochemistry**. Expression of HD5 in crypt epithelium and aldolase-B in villus epithelium is shown by immunohistochemistry analysis. HD5 **(A) **and aldolase-B **(B) **protein expression were evaluated by immunohistochemistry using peroxidase detection of mouse monoclonal anti-HD5 and anti-aldolase-B antibodies in consecutive serial tissue sections. Expression of HD5 was detected exclusively in Paneth cells, while aldolase-B was observed primarily in the epithelial cells of the villus region, providing supportive protein level evidence for the transcriptional differences elucidated by microarray and RT-PCR based assays.

To provide further support that the transcriptional profiles detected by microarray analysis accurately represented the differences between villus and crypt epithelial cells, we evaluated expression of selected genes by immunohistochemistry. In agreement with transcriptome assessments and previous studies [25,26], we detected the antimicrobial peptide HD5 exclusively in Paneth cells within crypt regions (Figure 4A). Aldolase-B, associated with carbohydrate metabolism in contrast, was detected primarily in epithelial cells lining the upper villus region within the same tissue sections (Figure 4B). These data served to both substantiate the corresponding microarray findings at the protein level, and provide further support for our interpretations of the global transcriptome data for crypt and villus epithelial cell populations.

**Figure 5:**
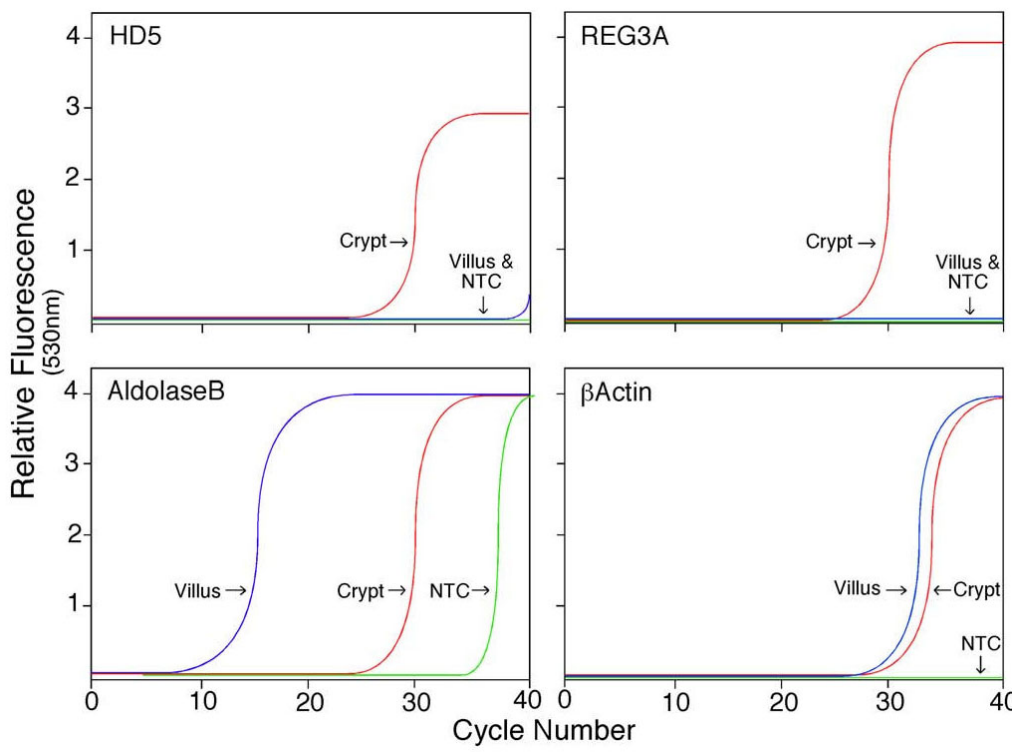
**Quantitative real-time PCR analysis of selected mRNAs in pools of villus and crypt epithelial cells**. Real-time fluorescence monitoring of PCR reactions using cDNA from villus and crypt samples obtained by LCMD. PCR primer pairs for HD5, Reg3A, aldolase-B or β-Actin were used in PCR reactions with cDNA templates from villus epithelial cells (VEC) or crypt epithelial cells (CEC). NTC, no template control. The cDNA was used as a template in real-time PCR analysis with specific primers (Table 1).

To further test if the microarray analysis accurately represented the differences in mRNA levels between villus and crypt epithelial cells, we evaluated expression of four genes by RT-PCR using the cDNA pools obtained by LCMD. Expression of HD5 and Reg3A mRNA was readily detected in the crypt epithelial cell cDNA pool, but was not evident in the villus epithelial cell cDNA under the same conditions (Fig 5, Crypt). By contrast, Aldolase B was detected in the villus epithelial cell cDNA sample (Fig 5, Villus) 15 cycles sooner than in the crypt epithelial cell cDNA sample, suggesting a difference in mRNA levels of approximately 2 × 10^4 ^fold. As a control, β-actin was detected in both cDNA pools at approximately the same levels. These data are consistent with the differential expression patterns of these genes detected by microarray hybridization analysis.

### Transcriptional profiling of ileal crypt epithelium

**Figure 6:**
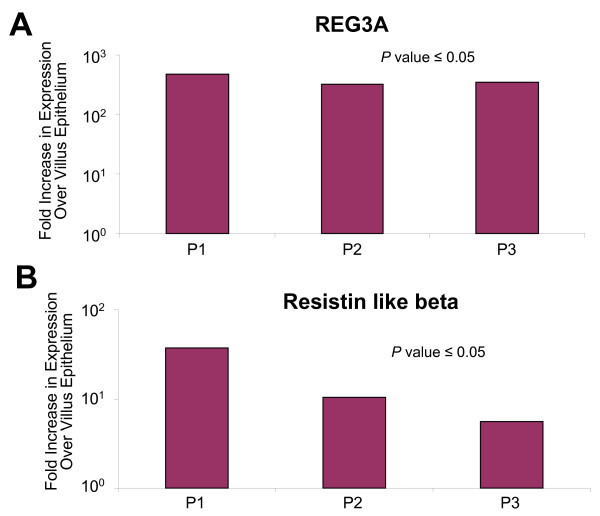
**Reg3A and RETLNB are transcribed exclusively in crypt epithelial cells**. Transcript levels of the anti-microbial, Reg3A **(A) **and a potential regulator of Reg3A, RETLNB **(B) **in crypt epithelial cells and villus epithelium of three ileal tissue samples. The mRNA levels of Reg3A were similar to the high levels measured for alpha-defensins, supporting a potentially important and orchestrated role for these proteins in innate defense responses in the human ileum. Fold differences in expression between the crypt and villus epithelial regions are show for 3 independent patient samples (P1, P2, and P3). Statistical validation of the fold-differences in mRNA levels was determined by student t-test (*P *value ≤ 0.05).

To elucidate potential mechanisms of Paneth cell function, we evaluated candidate genes from the microarray data that were enriched in the crypt epithelial cell pool and believed to be associated with immune responses, cellular growth, differentiation, and motility. One striking example, Reg3A was transcribed, on average, at >4000 fold higher levels in crypt epithelial cells than measured in villus epithelial cells (Figures 5 and 6A). Reg3A has been shown previously to be expressed in intestinal Paneth cells [22-24] and has been shown to have both anti-inflammatory and antimicrobial properties [24]. Our findings indicate that Reg3A is constitutively transcribed at substantial levels, similar to HD5 and trypsin in Paneth cells of the human ileum. These data suggest that in the healthy human ileum, Reg3A may function predominantly in regulating bacterial colonization.

Increased resistin-like beta (RETNLB) transcription was also detected in crypt epithelial cells (Figure 6B). In the colon, RETNLB is synthesized in both goblet and crypt region epithelial cells and appears to govern susceptibility to colonic inflammation [27]. Previous studies also suggest that RETNLB may regulate expression of Reg3A [27]. Thus, the coincident high level expression of RETNLB and Reg3A in crypt cells indicates that these molecules may function coordinately in the human ileum to mediate homeostasis of the commensal microbiota and/or attenuate inflammatory responses.

**Figure 7:**
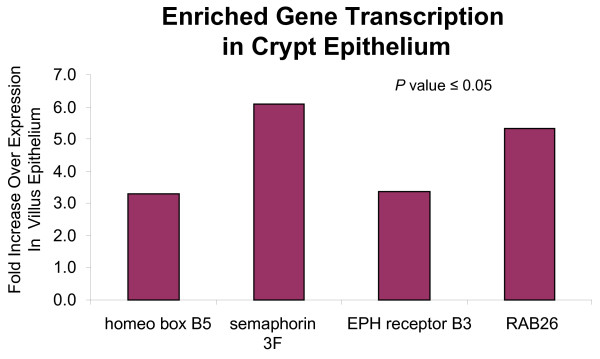
**Identification of crypt epithelial cell genes associated with differentiation, positioning, and secretory functions**. Four genes expressed in crypt epithelial cells, but not in villus epithelium, and potentially involved in epithelial cell maturation (homeobox B5), positioning (ephrin receptor B3, semaphorin F), and granule secretion (Rab26) were identified through microarray based transcriptional profiling. The lack of expression of these genes in villus epithelium may indicate that they function in concert exclusively in the Paneth cell lineage to promote cell-specific differentiation and migration patterns that lead positioning at the bottom of crypts and antimicrobial peptide secretion. Statistical validation of the fold-differences in mRNA levels was determined by student t-test (*P *value ≤ 0.05).

Other genes transcribed preferentially in crypt epithelial cells included several involved in epithelial growth and positioning that may represent a distinct set of biomarkers specific to this cellular compartment (Figure 7). These included EPH receptor B3, homeobox B5, and semaphorin 3F, implicated in previous studies to be involved in either the migration or specification of intestinal epithelial stem cell population. Ephrin B3 and its receptor are believed to be regulated by Wnt and expressed in crypt epithelia where they control the proper segregation of Paneth cells from post-mitotic cells [28,29]. Homeobox B5 (HOXB5) has been shown to regulate enteric development [30], while several investigations have highlighted the role of semaphorin 3F in inhibiting cell proliferation and migration [31,32]. It is noteworthy that we detected the stem cell marker, HOXB5, as this finding supports a recent report that stem cells may dispersed along both sides of the crypt base [18] and suggests that HOXB5 may be a biomarker of stem cells residing in the human ileum. Taken together, these data highlight a substantial presence of intestinal stem cells within the human ileal crypt, and suggest that EphB3, HOXB5, and SEMA3F may function in concert to regulate differentiation of epithelial progenitor cells and control the numbers and/or positioning of small intestinal Paneth cells.

### Transcriptional programs in villus epithelial cells

**Figure 8:**
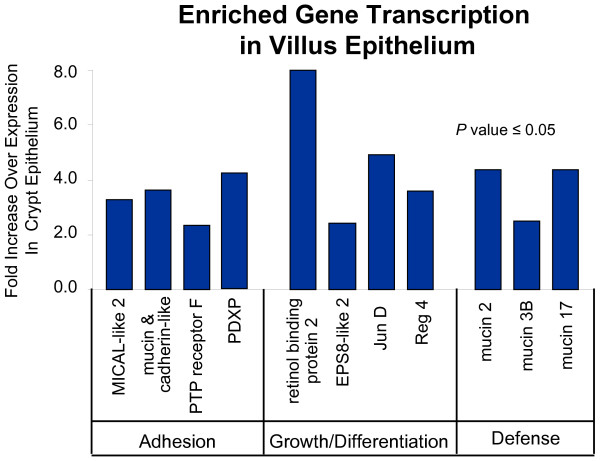
**Transcriptional profile of genes associated with villus epithelial cell maturation, adhesion, and defense response**. Multiple genes involved in epithelial cell adhesion, growth and maturation, and defense response were found to be transcribed exclusively in the villus epithelium. Interestingly, the genes involved in epithelial adhesion and differentiation were distinct from those identified in Paneth cells, suggesting that each lineage employs a separate and specific set of molecular mechanisms to promote appropriate growth, development, and positioning. Increased transcription of multiple mucins transcripts in the villus epithelium was also detected, an expected result given the observed level of goblet cells in tissue sections of the ileal samples that were used for LCMD. Statistical validation of the fold-differences in mRNA levels was determined by student t-test (*P *value ≤ 0.05).

Epithelial cells isolated from the intestinal villi displayed increased transcription of a set of genes associated with epithelial cell differentiation (Eps8-like 2, RBP 2, JunD, and Reg 4), adhesion (mucin and cadherin-like, protein tyrosine phosphatase receptor F, and MICAL-like 2), motility (pyridoxal phosphatase/PDXP), and cytoprotection (mucins 2, 3A, and 3B, major vault protein) that was distinct from the set detected in crypt epithelial cells (Figure 8). Elevated mucin expression in the villus epithelial cell population was expected and supported by the large number of goblet cells that typically populate ileal villi (Figure 1). In addition to the increased mucin, we detected enriched expression of MHC class IA, IC, and IF molecules and Fc fragment of IgG binding protein (data not shown), potentially indicative of the presence of intraepithelial lymphocytes within the epithelial lining. These findings were consistent with the likelihood that several epithelial cell types known to reside in the villus region were isolated by LCMD.

### Transcriptional patterns of ileal epithelial biomarkers along the GI tract

**Figure 9:**
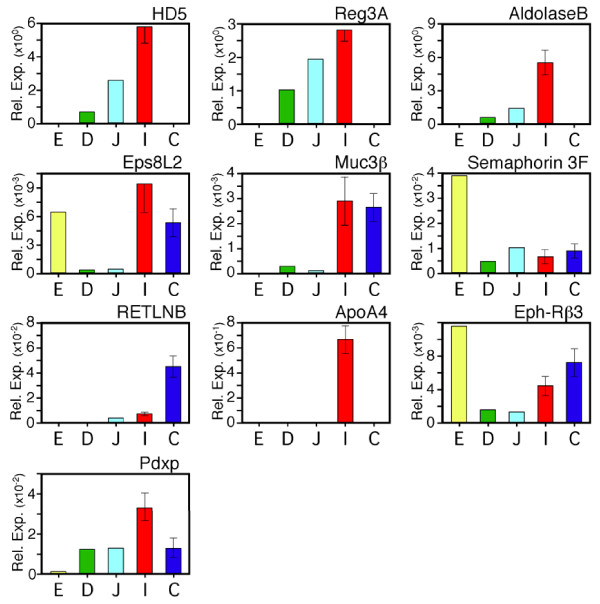
**Quantitative real-time PCR analysis of selected mRNAs in human gastrointestinal tissues**. Genes were selected for analysis from those that showed differential crypt-villus expression by microarray analysis, including mRNA encoding HD5, Reg3A, aldolase-B, Eps8L2, Muc3β, semaphorin3F, RETLNB, ApoA4, Eph-Rb3 and Pdxp. The mRNA copy counts were determined from standard curves using gene-specific plasmids and are expressed as a fraction relative to measure values for β-actin. Ileal samples (n = 5) and colon (n = 5) were from non-diseased surgical samples of adjoining tissue resected for bowel obstruction or colon cancer. Single samples of commercially obtained (pooled) RNA were analyzed for the other tissues. Error bars represent standard deviations from the geometric mean. For single specimen samples (E, D, J) inter-assay variation was <15% for mRNA copy counts ≥10 exp4/10 ng RNA, and 40% for mRNA copy counts ≤10 exp2/10 ng RNA. E, esophagus; D, duodenum. J, jejunum; I, ileum; C, colon.

To determine if the differentially expressed genes along the crypt-villus axis in the ileum are expressed at other regions of the gastrointestinal tract, we compared transcript levels of selected genes in the mucosa of the esophagus, duodenum, jejunum, ileum, and colon by RT-PCR (Figure 9). Villus epithelial cell biomarkers were also expressed at several locations along the gastrointestinal tract, yet, all appeared to be more highly transcribed in ileal tissues than at other sites. Eps8L2 and Muc3B transcripts were detected in the ileum and colon, while aldolase-B was almost exclusively detected in the ileum.

Interestingly, we found that Paneth cell markers HD5 and Reg3A were highly expressed in the ileum and moderately expressed in the jejunum, but not in the colon, or esophagus. A low level of mRNA, well below ileal and jejunal levels, was also detected in the duodenum, indicating that these antimicrobial peptides may function primarily in the more distal regions of the small bowel, consistent with the distribution of Paneth cells in the intestinal tract. In contrast, RETNLB and EphRB3 were transcribed predominantly at other sites, including the colon (RETNLB and EphRB3) and esophagus (EphRB3 only).

## Discussion

While LCMD and gene expression analyses have been employed successfully in studies of the adult mouse model and intestinal epithelial cell lines to identify mechanisms of function, differentiation, and positioning [3,4,19,21,28], to our knowledge the current report represents the first such *in vivo *investigation of the human small intestinal crypt-villus axis. It is notable, therefore, that many of the patterns of transcription observed in the villus and crypt epithelium pools that we identified here were in agreement with data from these previous reports. The consistency of these data with previous studies both underscored the reproducibility of the methodologies used for LCMD and mRNA detection in paraformaldehyde-fixed samples, and supported the overall accuracy of transcriptome data generated by microarray analysis. While it is likely that the integrity of mRNA isolated from paraformaldehyde fixed specimens is inferior to fresh-frozen specimens, we have found that short, overnight fixation protocols yield RNA of sufficient quality to perform consistent and reproducible RT-PCR and microarray analyses. However, due to the small sample sizes generated by LCMD, it was not feasible to check the RNA integrity by gel methods before proceeding to amplification and labeling. Thus, although the data presented here serves to illustrate the general feasibility and robustness of LCMD-based studies of paraformaldehyde fixed tissues, it remains possible that some small, but significant, differences between two cell pools may not be optimally detected by these methods and criteria.

Although high baseline transcription levels of alpha-defensins, lysozyme, and secretory phospholipase A2 in mammalian small intestinal Paneth cells have been documented [11,33,34], we were intrigued by the apparently similar level of transcription of the multifunctional lectin-related protein Reg3A. Reg3A transcription also displayed a markedly similar tissue specific expression profile to HD5 (Figure 5B), apparently transcribed along the alimentary tract exclusively within the small bowel. Like HD5 [16], Reg3A is known as a downstream Wnt/beta-catenin signaling target [35]. The murine ortholog of Reg3A (named Reg3γ in mice), is expressed in Paneth cells [23]. While this localization is notable in light of studies demonstrating its antimicrobial properties [23,36], Reg3A has also been shown to stimulate liver regeneration and growth [37]. Thus, it is possible that in the small intestine, high basal levels of Reg3A expression may act bifunctionally to contribute to rapid and robust local antimicrobial functions when required, and perhaps to promote epithelial cell differentiation or growth.

Potentially important cellular pathways employed by Paneth cells to enable rapid secretion of antimicrobials may have been discovered within the transcriptional profiles of the crypt epithelium, including enriched expression of the Ras-like GTP binding protein, Rab26. Although little is yet known about Rab26 function, the few studies that have been completed suggest it may be involved in mediating the transport of secretory granules in parotid acinar cells of the rat [38,39]. These investigations showed that Rab26 is exclusively associated with mature secretory granules that are recruited to the plasma membrane in response to increased Ca^++ ^influx induced by beta-adrenergic stimulation. Given these findings, we suggest that Rab26 may function similarly in ileal crypts, where release of secretory granules rich in antimicrobial peptides is a predominant physiological event.

Absorptive enterocytes and goblet cells are the predominant cell types of the villus epithelium (Figures 1A and 4). Our observation of considerable goblet cell numbers lining the villus epithelium in the ileal tissue samples utilized for LCMD (Fig 1) was reflected by increased mRNA levels of mucin 2 and mucin 3B (supplemental_microarray_data.xls). Among the transport associated genes we found enriched in the villus epithelium were several members of the solute carrier family (supplemental_microarray_data.xls). It is notable that this list included the oligopeptide transporter SLC15A1 and the amino acid transporter SLC7A7, both recognized as villus specific biomarkers, and shown to be expressed at high levels in the mouse ileum [3]. While much of the previous data on SLC15A1 and SLC7A7 have been generated from transcriptional profiling of whole tissue to determine the relative distribution of SLC transporters along the gastrointestinal tract, our findings extend these studies to confirm the presence of these molecules in the absorptive epithelial regions of the human ileum. In addition to SLC15A1 and SLC7A7, we detected enriched expression of the sodium/hydrogen exchanger SLC9A3 and the sulfate transporter SLC26A2 in the villus epithelial cell pool. These findings are potentially important in understanding how different absorptive/digestive processes may be organized along specific regions of the human small intestine, and their relationship to similar processes in animal models.

Villus epithelial cells also displayed clear increases in the expression of several growth and differentiation associated genes, and genes involved in establishing and maintaining epithelial cell polarity, as compared to the levels observed in crypt epithelial cells. Of the genes involved in epithelial growth, it was interesting to note that many may function through EGF signaling; a pathway enriched in the microarray data (Figure 2). Among the enriched genes in the villus epithelium controlling growth and differentiation, Reg4 was particularly interesting due to its role in activating the EGF receptor [40], and its classification, like Paneth cell Reg3A, within the regenerating (Reg) family of genes [41]. Thus, the large discrepancy between mRNA levels of Reg4 in the villus epithelium and Reg3A in crypt epithelium highlights that differential expression of this gene family may serve as useful indicators (perhaps even important mediators) of differentiation in human ileal epithelial cell lineages.

## Conclusion

Our study provides a comprehensive evaluation of gene expression patterns that characterize villus and crypt epithelial cells in healthy human ileal tissue. In addition to detecting biomarkers previously identified *in vitro *and in animal models, we have gained insight into the predominate physiological processes occurring in these regions and identified a distinct set of genes believed to be linked to epithelial cell motility, adhesion, and differentiation in each population. As such, these data provide a framework upon which future investigations of human small intestinal epithelial development and function can be further explored. Moreover, improved knowledge of biomarkers specific to human epithelial cell lineages in the ileum will also aid in the design and interpretation of studies on enteric diseases, including infectious enteritis and Crohn's disease. In addition, the ability to accurately detect and analyze gene expression in isolated cell populations from formalin-fixed specimens represents a potentially valuable new tool to further our understanding of intestinal epithelial cell differentiation and function beyond the scope of animal models.

## Methods

### Tissue specimens

Surgical specimens of intestinal mucosa were obtained from individuals undergoing surgery for colon cancer, bowel obstruction, or other non-inflammatory conditions. These samples were obtained through the Cooperative Human Tissue Network (funded by the National Cancer Institute) with Institutional Review Board approval, and in accordance with the Helsinki Declaration. Other investigators may have received specimens from the same subjects. Immediately after surgery, the mucosa was separated from the adjacent tissue. The specimens were free of macroscopic signs of disease or inflammation. The mucosal tissue was either snap frozen in liquid nitrogen and stored at -80°C for subsequent RNA isolation, or fixed overnight in 4% (w/v) paraformaldehyde, dehydrated in a graded alcohol series and paraffin-embedded.

### Cell isolations by LCMD

Epithelial cells from the terminal ileum were captured by LCMD from unstained 6 μm thick paraformaldehyde-fixed paraffin-embedded sections. All sectioning and subsequent specimen handling procedures were conducted under RNase free conditions. Sectioning was performed within two weeks of the LCMD procedure and cells were captured within one hour of deparafination. At least 500 epithelial cells of each type from each sample were laser catapulted using a PALM MicroLaser System (Zeiss, Oberkochen, Germany) into microcentrifuge tubes containing RNA extraction buffer. Total RNA was immediately extracted for microarray-based and real-time PCR-based gene expression analysis. All solutions and plasticware for these procedures were documented to be free of RNases.

### RNA isolation and reverse transcription for PCR analysis

Frozen tissue specimens were disrupted mechanically in guanidine thiocyanate buffer, and total RNA was isolated using cesium chloride gradient ultracentrifugation as described [42]. RNA was quantitated in duplicate using ultraviolet absorption spectrometry at 260 nm (Nanodrop Technologies, Wilmington, DE). For cDNA synthesis, 5 μg of total RNA was reverse transcribed with Superscript II reverse transcriptase (50 units, Invitrogen, Carlsbad, CA) using an oligo-(dT)12–18 primer as described [43]. The single-stranded cDNA product was purified using column adsorption chromatography (Qiagen, Valencia, CA). A single cDNA preparation from each specimen was used for each assay. Alternatively, for 3'-rapid amplification of cDNA end (RACE) analysis 5 μg of total RNA from human small intestine was reverse transcribed using the RACE/anchor primer 5'-CCATCCTAATACGACTCACTATAGGGCTCGAGCGGC(T)_18_(GCA)(GCAT)-3' as described previously [44]. The resulting cDNA product was used as a template in a PCR reaction using as gene specific primer common to both Reg3A and Reg3g 5'-CGCTGTCCCAAAGGCTCCAAG-3', and the antisense RACE primer 5'-CCATCCTAATACGACTCACTATAGGGC-3'. The reaction product was isolated and then subcloned. Ten independent RACE clones were isolated and DNA sequence analysis showed all corresponded to Reg3A.

### Quantitative real-time PCR

Quantitative real-time PCR using external standards was performed with gene-specific oligonucleotide primer pairs (Table 1) in a temperature cycler equipped with a fluorescence detection monitor (LightCycler, Roche Diagnostics, Mannheim, Germany), as described [43]. An aliquot of the cDNA for each sample (corresponding to 10 ng RNA) was used as a template in real-time PCR analysis with specific primers (Table 1). Absolute mRNA copy count determination was made per 10 ng total RNA for each gene, and the levels for each gene were expressed relative to absolute beta-actin levels, which were 4.7 × 10^4 ^for esophagus, 1.4 × 10^4 ^for duodenum, 1.1 × 10^4 ^for jejunum, 5.0 × 10^4 ^for ileum, and 0.6 × 10^4 ^for colon.

**Table 1:** Oligonucleotide sequences used in quantitative real-time RT-PCR analysis.

PCR Primer Pairs		
	Sense	Antisense

HD5	5'GCCATCCTTGCTGCCATT 3'	5'GCTTCTGGGTTGTAGCCTCATC 3'
REG3A	5'CGCTGTCCCAAAGGCTCCAAG 3'	5'GCACAGACACCAGGTTTCCAGAGG 3'
RELMB	5'CAGTGTTCCTTAGACTCCGTTATGG 3'	5'ACGGTCTGCCTTGGCTTTTG 3'
EPS8L2	5'CGGCTGGGCAAGAAGATGCG 3'	5'GTGCTGGAAGGGGATGGGCG *3'*
MUC3B	5' TGACGCTCAGCAAAACCGATAAC 3'	5' AACAGGCTTCAGGACCAAGACAGC 3'
PDXP	5' ACTCATCAATGTTGGGTCCTGTGG 3'	5' AGGGTCATCGTCACTTAGGCTTCC 3'
APOA4	5' AACTCACCCAGCAACTCAATGCC 3'	5' CTCCTTCCCAATCTCCTCCTTCAG 3'
SEMA3F	5' GCTGGTCAGAGACGGCAGAAAAC 3'	5' TGCTCCCTCCCTTTGTCCTGTAG 3'
EPHB3	5' AACGATGGGCAGTTCACGGTC 3'	5' GCTGTTGACAAGGATGTTGCGAG 3'
ALDOB	5' TGCTATCAACCTTTGCCCTCTACC 3'	5' CCCCAGAAGAACCCGTGTGAAC 3'
BActin	5' TGATGGTGGGCATGGGTCAG 3'	5' CGTGCTCGATGGGGTACTTCAG 3'

### Immunohistochemistry

Expression of HD5 and Aldolase-B was detected, at the protein level, by immunohistochemistry analysis using mouse monoclonal antibodies to HD5 [45] and aldolase-B (Dako, Carpinteria, CA). Sections were deparaffinized, hydrated in a graded series of alcohols, and washed in PBS pH 7.4. The sections were blocked with 5% normal goat serum, and then incubated in the mouse monoclonal antibody (dil 1:100) for 16 hours at 4°C in a humidified chamber. Sections were again washed in PBS, blocked with goat serum, followed by incubation with biotinylated goat anti-mouse (dil 1:200; Vector Laboratories, Inc., Burlingame, CA) for 20 minutes at room temperature. 5% normal goat serum was used as diluent for antisera. The sections were then treated with streptavidin-HRP complex and incubated with DAB substrate (Dako, Carpinteria, CA) for 8 minutes at room temperature, followed by light hematoxylin counterstain.

### Microarray analysis

Amplification of mRNA was performed utilizing protocols and reagents in the Paradise Reagent System (Molecular Devices, Inc. Sunnyvale, CA). Labeling, hybridization to Human X3P Array GeneChips^© ^(Affymetrix, Santa Clara, CA) staining and scanning were performed according to protocols in the Affymetrix Gene Expression Analysis Technical Manual. Labeled cRNA was hybridized for 16 hr at 45°C. The fluorescence emitted at 570 nm was measured and used to calculate and compare the expression level of each gene.

Stringent statistical criteria was applied to the analysis of microarray data using robust multi-chip analysis (RMA) based (Array Assist^©^) and model-based (dChip) algorithms [46]. A minimum 2-fold difference between mean levels of transcription in villus and crypt epithelial cells was used as criteria for determining "enriched" expression in each population. Fold differences were calculated from comparison of the mean transcription levels among at least 3 biological replicate samples (patients) of each epithelial region, with 95% confidence (*P*-value ≤ 0.05) by standard student t-test. Genes meeting fold-change statistical criteria were subjected to hierarchical clustering to identify common patterns of up- and down-regulated transcription, followed by comprehensive functional and statistical analysis of the biological pathways and processes represented by the genes in each sub-cluster. The Affymetrix GeneChip Operating System data files (*.cel, and *.chp) have been deposited in and made available to the public at the Gene Expression Omnibus database (accession number: GSE10629).

### Biofunctional analysis of microarray data

Assignment of genes to functional categories was performed through annotation of gene lists using the Affymetrix NetAFFX web interface, the DAVID annotation and biological process analysis tool [47], and through literature-based classification manually. Statistically over-represented (Fisher exact probability score < 0.05) biological processes within sub-clusters were identified using DAVID and Pathway Architect (Stratagne/Ioboin) analysis software. Following elucidation of biologically enriched themes, the microarray data was re-mined and analyzed with focus on identifying and interpreting all statistically valid changes gene expression associated with the highlighted pathways.

## Authors' contributions

MDG carried out the laser capture microdissection, performed gene expression analysis and immunohistochemistry, and drafted the manuscript. JW participated in the design of the study and performed laser-capture microdissection. RJK performed the RT-PCR analysis, CML participated in the study design and laser capture microdissection, SS performed the RT-PCR analysis, IG performed mRNA amplification and labeling, SD participated in coordination and design of the study and reviewing the manuscript, CLB conceived of the study, participated in its design and coordination, and helped to draft the manuscript. All authors read and approved the final manuscript.

## Supplementary Material

Additional file 1 Excel spreadsheet of genes in the functional categories listed in Fig 2 showing differences in transcription ≥ 2-fold between villus and crypt epithelial cells (*P *≤ 0.05). The data shows both the fold differences in expression and the statistical *P *values that correspond. Click here for file
